# Real-time, volumetric imaging of radiation dose delivery deep into the liver during cancer treatment

**DOI:** 10.1038/s41587-022-01593-8

**Published:** 2023-01-02

**Authors:** Wei Zhang, Ibrahim Oraiqat, Dale Litzenberg, Kai-Wei Chang, Scott Hadley, Noora Ba Sunbul, Martha M. Matuszak, Christopher J. Tichacek, Eduardo G. Moros, Paul L. Carson, Kyle C. Cuneo, Xueding Wang, Issam El Naqa

**Affiliations:** 1 Department of Biomedical Engineering, University of Michigan, Ann Arbor, MI USA; 2 Department of Machine Learning, Moffitt Cancer Center, Tampa, FL USA; 3 Department of Radiation Oncology, University of Michigan, Ann Arbor, MI USA; 4 Department of Nuclear Engineering, University of Michigan, Ann Arbor, MI USA; 5 Department of Radiation Oncology, Moffitt Cancer Center, Tampa, FL USA; 6 Department of Radiology, University of Michigan, Ann Arbor, MI USA

**Keywords:** Medical imaging, Biomedical engineering, Ultrasound, Translational research

## Abstract

Ionizing radiation acoustic imaging (iRAI) allows online monitoring of radiation’s interactions with tissues during radiation therapy, providing real-time, adaptive feedback for cancer treatments. We describe an iRAI volumetric imaging system that enables mapping of the three-dimensional (3D) radiation dose distribution in a complex clinical radiotherapy treatment. The method relies on a two-dimensional matrix array transducer and a matching multi-channel preamplifier board. The feasibility of imaging temporal 3D dose accumulation was first validated in a tissue-mimicking phantom. Next, semiquantitative iRAI relative dose measurements were verified in vivo in a rabbit model. Finally, real-time visualization of the 3D radiation dose delivered to a patient with liver metastases was accomplished with a clinical linear accelerator. These studies demonstrate the potential of iRAI to monitor and quantify the 3D radiation dose deposition during treatment, potentially improving radiotherapy treatment efficacy using real-time adaptive treatment.

## Abstract (web summary)

Radiation dose delivery is measured in real time for a patient with cancer.

## Main

Radiation therapy (RT) has been shown to improve the outcomes of patients with cancer and provide palliation of related symptoms^1^. Successful RT is contingent on delivering intended sufficient radiation dose to tumor while sparing surrounding normal tissues^2^. Achieving such a desired therapeutic ratio, that is, maximizing tumor control while minimizing toxicity, requires that the planned radiation dose is delivered accurately^3,4^.

To improve the efficacy of RT, advanced image-guided delivery technologies have been proposed and developed over the past decades^5,6^. Technologies such as intensity modulated RT and volumetric modulated arc RT can offset some of the limitations associated with three-dimensional (3D) conformal RT^7,8^; however, targeting of moving lesions remains challenging. Several studies have highlighted discrepancies between planned and delivered RT and their impact on tumor control^9^. These differences are exacerbated by setup errors, organ motion, as well as anatomical deformations^10,11^, which may markedly alter the intended doses delivered to the target or adjacent normal tissues over the course of treatment^12–14^. Currently, the common practice for creating a planning target volume (PTV) is to expand the clinical target volume with a spatial margin to allow for setup uncertainties and organ deformations^15^. Moreover, dose escalation in many diseases is limited by adjacent normal tissue radiosensitivity^16,17^. In the case of patients with liver cancer, a previous study showed reducing the margin for organ motion can reduce the effective treatment volume by up to 5% (resulting in a reduced complication risk of 4.5%), which would allow escalation of radiation dose by 6–8 Gy, resulting in improved tumor control by an estimated 6–7% (ref. ^18^).

To mitigate problems with target and normal tissue motion, technologies capable of monitoring tumor location and mapping of the delivered dose during treatment are required. Surrogates of motion such as fiducials^19^ or active breath hold with spirometry are sometimes used for respiratory gating^20^. In addition, several onboard image-guidance RT (IGRT)^21,22^ technologies have been used, including electronic portal imaging device^23,24^, kilovolt fluoroscopic imaging and kilo- or megavolt cone beam computed tomography (CT) (CBCT) imaging. However, none of these technologies can provide real-time information of the 3D dose deposition. Safer nonionizing technologies were also explored, such as ultrasound imaging^25^ and surface camera-based systems, which are susceptible to subtle sources of error and interuser variability. To better resolve tissue discrimination with real-time imaging, integrated technologies such as CT-linear accelerators (LINACs), magnetic resonance imaging- (MRI-) LINACs and positron emission tomography-LINACs have been introduced for clinical use^26^, but CT, MRI or positron emission tomography cannot monitor the location of the X-ray radiation beam nor the dose deposition in the normal tissues or the target. Currently, image guidance with delivered dose feedback monitoring remains inherently limited^27^. On the other hand, there are a wide variety of devices for clinical dose measurements (for example, diodes, thermal/optical stimulated dosimeters, metal oxide semiconductor field effect transistors, plastic scintillators, electronic portal imaging devices, gels and films). These devices, however, are mostly limited to point measurements on the external surface of a patient and are not volumetric, not real time and some are dose rate or energy dependent^28^. New generations of detectors can be used in vivo but do not provide any of the necessary detailed anatomical information^29–31^. Therefore, there is a long-standing clinical need for more effective imaging technologies capable of volumetric, real-time, in vivo dose delivery monitoring during RT for feedback guidance.

Ionizing radiation acoustic imaging (iRAI) is a noninvasive imaging technology that reconstructs the radiation dose using acoustic waves stemming from the absorption of pulsed ionizing radiation beams in soft tissue^32,33^. iRAI has the potential to map the dose deposition and monitor the dose accumulation at in-depth anatomical structures in real time during RT. In contrast to other dose mapping methods, iRAI is directly proportional to the radiation dose absorbed by the targeted tissue. With precalibration of the Grüneisen parameter, medium density, pulse time profile and sensor sensitivity, the linear relationship between the absorbed dose and deposited dose could enable iRAI to both localize and quantify the absolute dose deposition during RT^32–37^. Most recently, the feasibility of iRAI for real-time monitoring of misalignment between the targeted tumor and the delivered beam has been presented for conventional as well as ultra-high dose rate (FLASH) radiation treatments^32,34,38^.

**Fig. 1:**
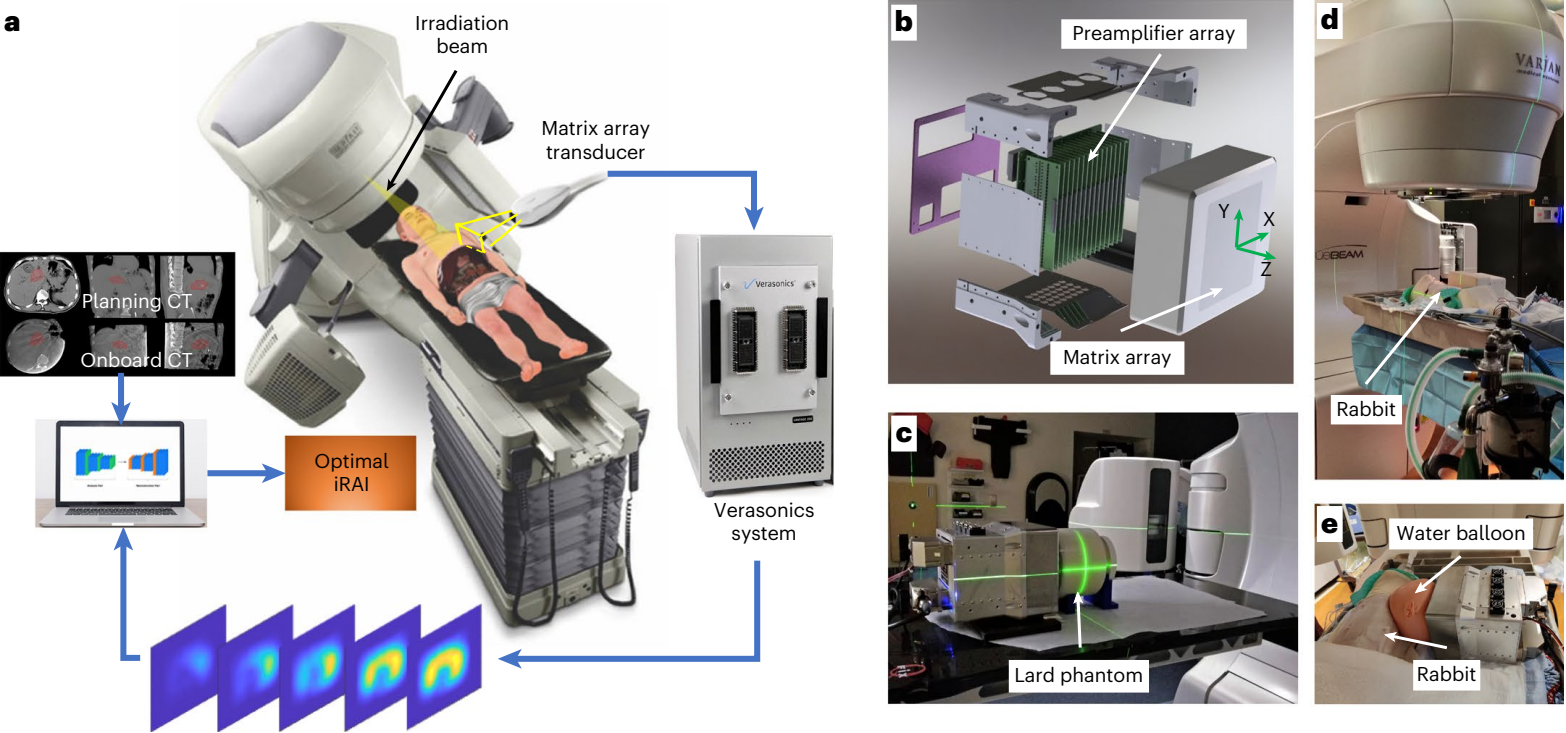
iRAI system schematic and the experimental setup. **a**, 3D schematic of the iRAI system for mapping the dose deposition in a patient during RT delivery. **b**, CAD view of a 2D matrix array with an integrated preamplifier board. The *xyz* coordinate system for the 3D iRAI imaging space is marked. **c**, The experimental setup for the phantom studies. **d**, The side view of the rabbit experiment setup in a clinical environment. **e**, Details regarding the transducer position and coupling of the rabbit experiment.

To further develop iRAI and promote its clinical translation, in this study we demonstrate a clinical ready iRAI system for real-time, volumetric imaging of radiation dose with high sensitivity and high spatial resolution, as shown in Fig. 1a. This imaging system was developed with a custom-designed two-dimensional (2D) matrix array transducer and a matching multi-channel preamplifier board (Fig. 1b), which were driven by a commercial research ultrasound system. Using this imaging system, iRAI was successfully performed with a lard phantom (Fig. 1c), an in vivo rabbit model (Fig. 1d,e) and patients with cancer undergoing radiotherapy on a clinical LINAC system. This study realized 3D semiquantitative mapping of X-ray beam delivery deep into the body during cancer treatment.

## Results

### iRAI system calibration

**Fig. 2:**
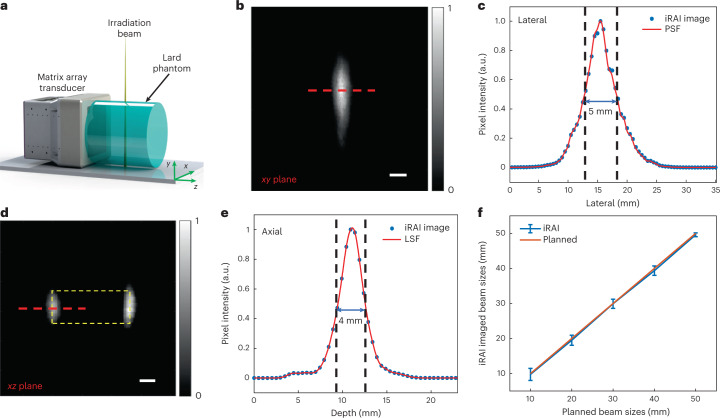
The performance of the 2D array transducer. **a**, Schematic of the iRAI phantom experiment for performance calibration. **b**, iRAI imaging with the 5 × 5 mm radiation beam field. Scale bar, 5 mm. **c**, Point spread function (PSF) of iRAI in lateral direction. **d**, Cross-section of iRAI imaging with 3 × 1 cm radiation beam field. Scale bar, 5 mm. **e**, LSF of the iRAI in the axial direction. **f**, Beam widths of iRAI versus the beam field sizes of radiation source along axial direction. Error bars are s.d. for *n* = 5 independent measurements.

Using the schematic setup shown in Fig. 2a, the iRAI result for a small field with a lateral plane on a cylindrical lard phantom is shown in Fig. 2b. The normalized intensity profile along the dotted line in the red box is presented in Fig. 2c, where the dots show the pixel intensities. The curve shows the fitted point spread function, which has a full-width at half-maximum of 5 mm, suggesting a lateral spatial resolution of roughly 5 mm. The cross-section of the iRAI result along the axial direction with a 1 × 3 cm beam is shown in Fig. 2d. Figure 2e shows the fitted line spread function (LSF) extracted from the front edge of the iRAI image with a 1 × 3 cm beam. The 4 mm full-width at half-maximum of the LSF suggests that the axial resolution of the 2D array is better than 4 mm, which is about the predicted theoretical resolution of our 350 kHz transducer. The iRAI detected beam sizes versus the beam sizes of the radiation beam along the axial direction are shown in Fig. 2f. For each delivered beam size, the mean and the standard deviation (s.d.) of the iRAI measurements are shown. A linear fitting was performed, and an *R*^2^ = 0.989 was achieved, demonstrating that the 2D array based iRAI imaging system can accurately measure the beam size with a maximum deviation of 1.75 mm and a mean ± s.d. of 1.25 mm.

### Mapping the dose distribution and temporal dose accumulation

**Fig. 3:**
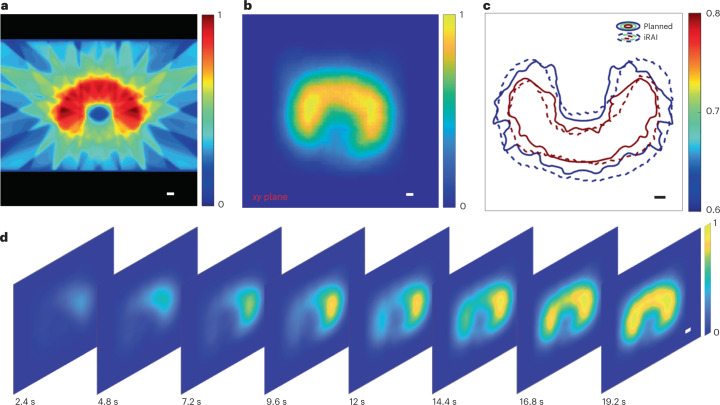
iRAI imaging for a C-shaped dose distribution treatment plan. **a**, The planned dose for the C-shaped 3D CRT treatment plan. **b**, iRAI imaging of relative deposited dose result for a C-shaped dose distribution treatment plan. **c**, The 60 and 80% isodose lines on the planned dose distribution and the iRAI imaged relative dose distribution. **d**, The temporal dose accumulation at different time points imaged by iRAI during the dose delivery of a C-shaped treatment plan. Scale bars **a**–**d**, 5 mm.

A C-shaped treatment plan with a dose distribution shown in Fig. 3a was delivered to a lard-based cylindrical phantom (Fig. 1c). The iRAI image showing the measured relative dose distribution in the phantom presents a C-shape, as shown in Fig. 3b. The planned dose distribution and the iRAI imaged dose distribution are compared in Fig. 3c, where isodose lines of 60% (blue) and 80% (brown) of the maximum dose are shown. There is good agreement in the shape of the 60 and 80% isodose lines between the planned dose and the iRAI imaged dose with an average root mean square error (r.m.s.e.) of 0.0987. A variation of less than 2% was achieved between the five independent iRAI imaging results, as shown in Supplementary Fig. 1 and Supplementary Video 1, which suggests that iRAI has high stability for measuring the dose deposition during RT.

With the C-shaped treatment plan, the Truebeam LINAC system (Varian) delivered the dose with 1,400 monitor units per minute. The temporal dose accumulation in the phantom over the delivery time of around 19 s was continuously monitored by iRAI, as shown in Fig. 3d. A gradually formed C-shaped dose distribution was clearly demonstrated by the iRAI image as a function of time with a 2.4-s interval. With averaging more than 100 pulses for iRAI image reconstruction, a frame rate of 3.3 Hz was achieved for monitoring the temporal dose accumulation in this study and is provided in Supplementary Video 2. The results for showing the delivered dose between two consecutive reconstruction time points are shown in Supplementary Fig. 2 and Supplementary Video 3. Since it typically takes around 60–120 s for a patient to receive one fraction of treatment, the iRAI system would be able to provide sufficient temporal resolution for monitoring dose delivery clinically.

### Mapping dose deposition of a treatment plan in an animal model

**Fig. 4:**
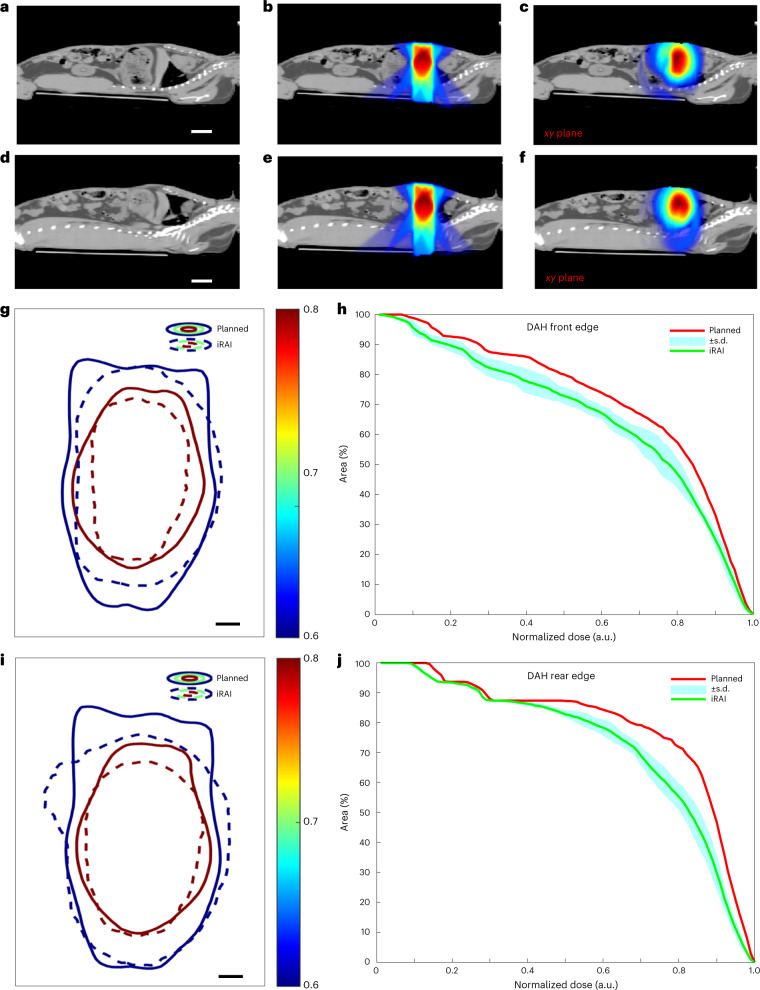
In vivo iRAI imaging versus the treatment plan of a rabbit model. **a**, The CT cross-section image of a rabbit in the front edge of the treatment dose delivery. **b**, The treatment plan fused onto CT the anatomy structure in the front edge of the dose delivery boundary. **c**, The iRAI image showing the dose distribution fused onto the CT scan at the same location of **b**. **d**, The CT cross-section image of the rabbit at the rear edge of the treatment dose delivery. **e**, The treatment plan fused onto the CT anatomy structure at the rear edge of the dose delivery boundary. **f**, The iRAI image showing the dose distribution fused onto CT scan at the same location of **e**. **g**, The 60 and 80% isodose lines of the iRAI measurement and the treatment plan in the front edge cross-section. **h**, The DVH of iRAI measurement in the front edge of the rabbit liver. The data with the blue areas are presented as mean ± s.d. for *n* = 3 independent iRAI measurements. **i**, The 60 and 80% isodose lines of the IRAI measurement and the treatment plan in the rear edge cross-section. **j**, The DVH of iRAI measurement in the rear edge of the rabbit liver. The data with the blue areas are presented as mean ± s.d. for *n* = 3 independent iRAI measurements. Scale bars in **a** and **d**, 2 cm; **g** and **i**, 5 mm.

Before the treatment planning simulation, the rabbit anatomy was obtained by CT scanning. The anterior CT cross-section image in the anterior plane of the front and the rear edges of the planned dose are shown in Fig. 4a,d, respectively. The definition of the front and rear edges is shown in the sagittal plane of the rabbit cross-section images in Supplementary Fig. 3. Fusion of the treatment planned dose distributions and the CT images at the same positions is shown in Fig. 4b,e, respectively. As shown in Fig. 4c,f, the front and rear edges of the iRAI images, which were extracted from the iRAI volumetric image based on the distance between the 2D matrix array and the isocenter of the treatment plan, were fused onto the corresponding CT images. By comparing the iRAI images and the treatment plan, the higher dose areas of the iRAI images were highly consistent with the plan, yielding an r.m.s.e. of 0.0570 and 0.0691 for the front and rear edges, respectively.

To further quantify the dose distribution, 60 and 80% isodose lines and a digital area histogram (DAH)^39^ from the iRAI result were compared to those of the treatment plan. As shown in Fig. 4g, the total distribution of isodose lines in the front edge of the iRAI image matched well with the treatment plan. Along the vertical direction, the iRAI image can resolve the same dose distribution as the treatment plan. Along the horizontal direction, the dose distribution presented by the iRAI result appears narrower than that of the treatment plan. Three independent iRAI measurements at the front edge were also quantified with DAH, as shown in Fig. 4h. The trend of the histogram percentage of the iRAI measurement is similar to the treatment plan. The blue area shows the s.d. of three independent iRAI measurements with a mean ± s.d. of 0.0199, which indicates that the iRAI imaging of deposited dose is stable. In addition, the rear edge isodose line shown in Fig. 4i has a consistent dose distribution in the bottom part. Although the top area shows some mismatch between the treatment plan and the iRAI results, overall, there is a good overlap agreement between the two distributions. The DAH results in Fig. 4j represent the relationship between three independent iRAI measurements and the treatment plan with a variation of less than 5%. iRAI measurements had a small s.d. of 0.0288. A slightly higher mismatch can be found with 70 to 90% of the maximum dose, which is also consistent with the isodose line results of Fig. 4i.

### Mapping dose deposition of a treatment plan in a cancer patient case

**Fig. 5:**
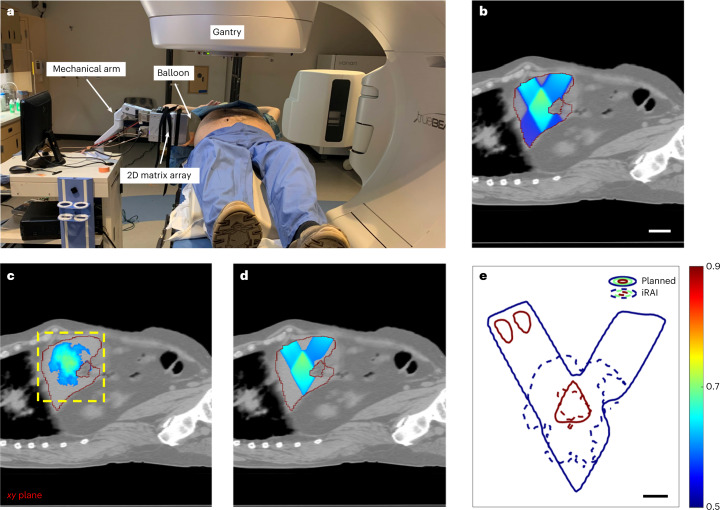
In vivo iRAI imaging versus the treatment plan on a patient. **a**, A photograph of the iRAI imaging on a patient taken during RT. **b**, The dose distribution of only the two static sagittal beams of the treatment plan with a liver mask fused onto the CT scan anatomy structure. Scale bar, 5 cm. **c**, The iRAI measurement of dose with a liver mask fused onto the CT anatomy structure with the same position as **b**. The yellow dashed box indicates the field of view of the 2D matrix array. **d**, Dose distribution (>50%) of the treatment plan with a liver mask fused on the CT anatomy structure. **e**, The 50 and 90% isodose lines in the iRAI measurement and the treatment plan. Scale bar, 2 cm. The red line in **b**–**d** indicates the boundary of the liver.

The clinical setting for performing iRAI imaging on a patient is shown in Fig. 5a. Due to the limited field of view of the 2D matrix array, only the radiation induced acoustic effects occurring in the liver were analyzed. As shown in Fig. 5b, a liver mask was applied to the treatment plan, which ensured that only the dose deposited to the liver was shown in the CT scan. The iRAI measurement results of the relative dose delivery of the two sagittal static fields are shown in Fig. 5c. The sagittal plane position of the iRAI image is shown in the sagittal plane of the patient’s cross-section images in Supplementary Fig. 4. Due to the limited signal-to-noise ratio (SNR), only the central part of the dose distribution was mapped by iRAI. The beam path of the two anterior beams was not resolved by iRAI. Taking into account the dose distribution of the treatment plan, doses lower than 50% of the maximum dose were removed from the treatment plan. This resulted in a diamond-shaped dose map, as shown in Fig. 5d. Comparing the iRAI measurement in Fig. 4c to the treatment plan in Fig. 5d, both the dose locations and the overall distributions are matched well. To further quantify the accuracy of the iRAI relative dose mapping, the 50 and 90% isodose lines were drawn based on the normalized dose in both the iRAI image and the clinical treatment plan^40^. The central two dose distributions matched well, especially for higher doses (90% isodose line), as shown in Fig. 5e. In addition, the 50% isodose line had relatively strong variation, only the central part around the target was imaged successfully by iRAI, which is reasonable considering the limited field of view of the 2D matrix array with an r.m.s.e. of 0.0787.

## Discussion

The goal of this study was to demonstrate a clinically applicable technique to increase the precision of in vivo dose monitoring during RT by mapping the dose deposition and resolving the temporal dose accumulation while the treatment is being delivered in real time. To achieve this goal, a clinical grade iRAI volumetric imaging system was developed. This was achieved by using a custom-designed 2D matrix array with a central frequency and bandwidth to match the spectrum of the acoustic wave induced by a 4-µs radiation pulse. This, together with the specially designed large size of the transducer elements, enhanced the sensitivity of detecting the weak radiation induced acoustic signal. To further improve the detection sensitivity, a custom-designed low-noise multi-channel preamplifier board was integrated with the matrix array for signal amplification before the signals are acquired by the research ultrasound system. This study has been able to detect the intrinsically weak thermoacoustic signal induced by the radiation beam in deep tissue such as the liver.

As demonstrated by the results, the C-shaped dose distribution can be imaged online using iRAI with high accuracy, while the iRAI measurements of the rabbit showed high consistency between the measured dose distribution and the one generated by the treatment planning system. Both in vitro and in vivo repeated stability measurements suggest that the iRAI system has high stability in mapping the delivered dose. In the patient study, despite the fact that the acoustic inhomogeneity of human tissues was neglected and the field of view of the 2D matrix array was limited here, the iRAI measurement clearly visualized a dose distribution similar to that of the treatment plan in vivo. Although the treatment plans for both the rabbit model and the patient are relatively simpler than common treatment planning procedures, the results from this study demonstrated that the iRAI is a clinically feasible and practical technique for real-time mapping of the 3D dose deposition during radiotherapy. By using state-of-the-art image processing and displaying technologies, iRAI volumetric dose measurement was achieved simultaneously during the radiation dose delivery of a deeply seated organ such as the liver. A continuously formed C-shaped dose during the radiation treatment shows a promising result for directly visualizing the dose accumulation of a treatment plan during delivery, which is an important step for establishing an online feedback system for RT active monitoring. To quantify the accuracy of iRAI for dose mapping, isodose line and DAH, which are two of the clinical standard quality assurance methods, were estimated for iRAI relative dose measurement^39,41^. The well-matched isodose lines of normalized iRAI measurement and the clinical treatment plan provide a proof of principle for the spatial accuracy of iRAI measurement in mapping the dose deposition in a clinical environment. The DAH results of iRAI measurement and the treatment plan in the rabbit liver show the same dose distribution, which also corroborate the accuracy of iRAI for relative 3D dose distribution mapping.

Despite the promising results achieved by the iRAI volumetric imaging system, there are still several limitations that could be addressed by future development of this technology. First, the sensitivity of iRAI in detecting the dose distribution should be improved. As demonstrated by our patient study, the high dose area can be mapped by iRAI volumetric imaging with high accuracy, while the lower dose intensity areas are still a challenge to image with the current system. Since multiple averaging is needed to achieve sufficient SNR for dose reconstruction, the current detection range is limited by the magnitude of the absolute dose delivered in the region of interest. To improve the detection sensitivity, not only the ultrasound array and the preamplifiers but also the system for signal digitization, processing, and image reconstruction should be further optimized. Second, volumetric dose distribution in deep tissues presented by the current imaging system is only semiquantitative, which provides relative dose measurements. The normalized color bar of each iRAI image indicates the relative dose instead of the absolute dose. To achieve iRAI imaging capable of providing absolute dose measurement, a protocol for comprehensive calibration is needed, which would consider the signal response of the imaging system, the temporal shape of the radiation pulse and the tissue properties (for example, physical density, speed of sound, coefficient of thermal expansion and specific heat capacity). This process has been demonstrated for photon and electron Cerenkov imaging with corresponding budget uncertainty and could be applied here too^42,43^. Specifically, for iRAI, the tissue properties are different for each individual, which, however, could be measured by the existing imaging methods such as CT, MRI and ultrasound, and information could be incorporated into the reconstruction algorithm using artificial intelligence methods^36,44,45^. Third, the spatial resolution of the current imaging system is still limited. As demonstrated by the quantified imaging results, the axial resolution and the lateral resolution of the current system are 4 and 5 mm, respectively. This spatial resolution, although already better than the clinical realistic accuracy of 5 mm^46^, can be further improved. To accommodate the low frequency of the acoustic signal produced by this 4-µs duration of the radiation pulse, the custom-designed matrix array works at a central frequency of 350 kHz. In the future, when working with a radiation beam with a shorter pulse duration, transducers with higher center frequencies leading to higher spatial resolution can be used. Fourth, the current iRAI system is a single-modality, and cannot enable pulse-echo ultrasound imaging at the same time. This is due to the limitation that the preamplifier board of the current iRAI system is receiving only and cannot transmit ultrasound pulses. Moreover, the central frequency of the current 2D array is only matched with iRAI acquisition and is unable to provide acceptable ultrasound imaging quality, which is typically in MHz range (roughly 1–3 MHz). In the future, powered by a well-designed preamplifier board and dual-frequency 2D matrix array enabling both receiving and transmission, iRAI and ultrasound volumetric imaging could be performed at the same time during RT so that both the 3D dose deposition and the tissue motion can be monitored simultaneously. Last, due to the limited bandwidth of the 2D matrix array, iRAI mostly images the edges of the radiation field, which also has consequences when aiming to assess the absolute dose delivery in 3D. Potential solutions can be learned from the well-developed photoacoustic imaging field by implementing better reconstruction algorithms and acquisition hardware^47,48^. In addition, as an ultrasound-based imaging modality, iRAI is applicable to ultrasound imaging compatible organs (for example, liver, breast, prostate and cervical) and shares the same limitations of ultrasound imaging within organs containing body cavities and bones.

In summary, this study describes an online iRAI volumetric imaging system that directly maps the dose deposition deep inside a human patient receiving a radiotherapy fraction without interrupting the treatment delivery. Despite the fact that both the sensitivity and the spatial resolution of iRAI could be further improved, the current system enabled these proof-of-concept experiments on phantoms, animals and especially human studies, demonstrating the feasibility of iRAI for clinical application during conventional RT by mapping the dose deposition for each treatment fraction. The iRAI system presented in this work also holds promise for applications in advanced RT modalities in online monitoring and accurate quantification of radiation dose deposition, such as real-time adaptive radiotherapy, FLASH RT and proton therapy.

## Methods

### iRAI acquisition system design

A clinically ready iRAI imaging system was adapted from our previous prototype iRAI and ultrasound dual-modality imaging system^32^, shown in Fig. 1a. To further improve the system sensitivity and add the volumetric imaging capability, the iRAI detector and amplification components were thoroughly redesigned to achieve real-time 3D imaging of deposited dose during RT. In this system, the radiation acoustic signals were detected by a custom-designed 2D planar matrix array (Imasonics, Inc.) with 32 × 32 = 1,024 (116.6 × 116.6 mm) elements, 3.45 × 3.45 mm element dimension and 0.2 mm kerf. The central frequency of 0.35 MHz, with 50% bandwidth, was chosen to match the power spectrum of the radiation acoustic signals generated by the approximately square, 4 µs X-ray pulse. This is crucial to enhance the SNR when detecting radiation acoustic signals so that highly sensitive dose mapping can be realized in real time. To further enhance the SNR, a custom-designed 1,024-channel preamplifier (AMP 1024-19-001, Photosound Technologies, Inc.) with 46 dB gain was fully integrated with the 2D matrix array, shown in Fig. 1b. This design avoided the cable connection between the transducer elements and the preamplifier and minimized the noise that could be introduced. The 2D matrix array with the integrated preamplifier board was driven by a 256-channel research ultrasound system with operation software v.4.4.0 (Vantage, Verasonics Inc.) via a 4 to 1 multiplexer, which was controlled by an Arduino microcontroller. The pulse trigger from the LINAC was precisely controlled by a delay generator and synchronized with the multiplexer and the ultrasound system. An acquisition process by the 1,024 channels was achieved for every four LINAC triggers. The iRAI images were displayed with 25 times averaging to further improve the SNR.

### iRAI system performance calibration

To verify the performance of the newly developed 3D iRAI imaging system based on the 2D matrix array, a resolution calibration with a 6-MV static beam from a clinical LINAC (TrueBeam, Varian Medical System Inc.) was performed. As shown in Fig. 1c, a cylindrical lard phantom in a 15 cm diameter plastic jar was made as a reference for calibration. The bottom part of the jar was removed and coupled with the surface of the 2D matrix array using ultrasound coupling gel. To calibrate the lateral resolution, a 5 × 5 mm radiation beam field was delivered by the LINAC, targeted to the middle of the lard phantom. The beam to array distance through the lard was approximately 10 cm. The axial resolution was calibrated through a front edge of a 1 × 3 cm beam using a LSF. To verify the performance of the system in measuring the size of the radiation beam in 3D, radiation beams with different sizes irradiated the phantom from above. The size of the beam along the lateral direction of the 2D array was kept at 1 cm, while the size along the axial direction was changed from 1 to 5 cm, with increments of 1 cm, shaped by controlling the multi-leaf collimator of the LINAC. Five independent iRAI volumetric images of different beam sizes were acquired for further statistical analysis.

### Mapping the dose distribution and temporal dose accumulation

To verify the feasibility of this imaging system in mapping the dose deposition and monitoring the temporal dose accumulation during a radiation treatment, a treatment plan with a C-shaped dose distribution was created, following a clinical protocol. The radiation treatment was on the same cylindrical lard phantom previously described. The 3D conformal radiation treatment (3D CRT), shown in Fig. 3a, consisted of 23 beam angles delivered with a maximum dose of 7 Gy by a TrueBeam accelerator (Varian Medical Systems) with 6-MV flattening filter free. During the radiation delivery, the isocenter of the treatment was aligned with the geometrical center of the phantom. Two different experiments were performed based on this C-shaped target treatment plan to evaluate both the dose distribution mapping and temporal dose accumulation monitoring. To assess the mapping of the dose deposition of each planned beam, the radiation induced acoustic signals were continuously acquired during the dose delivery and then processed by a delay-and-sum image reconstruction algorithm via MATLAB 2020a (Mathworks). Once the dose delivery was completed, the acquired acoustic signals from each beam were combined coherently by summing the signals from each pulse and each element to form an iRAI image for the whole treatment plan. An envelope was formed along the normal direction of the 2D matrix array after the delay-and-sum reconstruction. Five independent iRAI volumetric image acquisitions of the same treatment plan were acquired for further statistical analysis. For monitoring the temporal dose accumulation, the iRAI image was reconstructed and displayed during the radiation beam delivery with an average of every 25 full acquisitions (equivalent to 100 radiation pulses). The online displayed image was shown in two formats: (1) total accumulated dose; and (2) the delivered dose between two consecutive reconstruction time points. Three independent iRAI volumetric images were acquired of the same treatment plan for further statistical analysis.

### Mapping dose deposition of a treatment in an animal model

Animal experiments were performed using a rabbit model to examine the feasibility of iRAI in mapping the dose deposition during RT in vivo with a clinical treatment plan. The photography of the imaging setup is shown in Fig. 1d. All the animal experiments were approved by University of South Florida Research Integrity and Compliance Institutional Animal Care and Use Committee (Combined Radiation Acoustics and Ultrasound Imaging for Real-Time Guidance in Radiotherapy, IS00008026). Two female New Zealand white rabbits (4.5–5 kg) of 6 months old, ordered from Charles River, were involved in this study. CT scanning (CT simulation) for these two rabbits was performed as input into the treatment planning system (Raystation 11A, RaySearch Laboratories). The treatment plan consisted of four 6-MV flattening filter free 3 × 3 cm beams at various gantry angles (30, 40, 320 and 340°) along the anterior plane of the rabbit with the liver placed at isocenter, consisting of a maximum dose of 5.36 Gy for each fraction.

During the experiment, anesthesia was induced using ketamine (40 mg kg^−1^) via intramuscular injection and maintained with 1.5% isoflurane and oxygen using a V-Gel (J1350D, Jorgensen Laboratories) and Matrx vaporizer (MidMark Corporation). Vitals (heart rate, respiratory rate, oxygen saturation and body temperature) were continuously monitored using a SurgiVet Advisor vital signs monitor (Smiths Medical) to ensure animal safety and to evaluate the anesthesia level. An adjustable water-circulating heating pad (TP-700, Stryker Corporation) was used to keep the body temperature stable. The 2D matrix array was directly facing the isocenter of the animals. The detection surface of the 2D matrix array was directly facing the isocenter and positioned parallel to the anterior plane of the rabbits, which was in the supine position with the head toward the gantry. A water-filled balloon was used for acoustic coupling between the rabbit abdomen and the array surface, as shown in Fig. 1e. The clearance distance between the isocenter and the array surface was 15 cm. A CBCT scan was performed before the treatment for image guidance during the positioning setup and, subsequently, three consecutive treatment fractions were performed to deliver the dose to the rabbit liver and imaged by iRAI for statistical analysis. Animals were euthanized right after the last treatment.

### Mapping dose deposition of a treatment plan in a cancer patient case

This human patient study was conducted to further evaluate the clinical feasibility of iRAI in mapping dose deposition in a treatment fraction. The study was approved by the institutional review board of the University of Michigan (UMCC 2017.160 Pilot Study of Combined Radiation Acoustics and Ultrasound Imaging for Guidance in Radiotherapy, HUM00139322). Informed consent was obtained after the nature and possible consequences of the studies were explained. A 60-year-old man diagnosed with liver metastasis was treated in this study. To minimize the interference for RT, the treatment plan for each fraction was divided into two parts. The first part was for iRAI imaging and consisted of 2.087 and 0.877 Gy beams delivered in the superior and inferior anterior directions, respectively. Two anterior beams with an angle of 60° formed a diamond-shaped dose in the central part of the liver, where the tumor was located. The second part was a volumetric modulated arc therapy (VMAT) plan to ensure that the total delivered dose met the clinical requirements. The 3D beam arrangements of the treatment plan are shown in Supplementary Fig. 5. Specifically, CT simulation included a 4D CT and a breath hold 40 s delay contrast scan. The contrast scan was fused with the 4D CT, and a gross tumor volume and internal target volume were made to include the respiratory motion of the tumor. A margin of 5 mm in the axial plane and 8 mm superior and inferior was applied to the internal target volume to make the PTV. The prescribed radiation dose was 54 Gy in total, delivered in three 18 Gy fractions to the PTV. The PTV volume receiving 100% of the prescribed dose (V100%) was 98.5% and the minimum dose to 100% of the PTV volume (D100%) was 90.1%. The treatment plan went through a standard optimization process. All standard organ at risk limits in the treatment plan directive were met. The beam arrangement consisted of one axial VMAT arc that delivered 89% of the prescription and two sagittal static fields that delivered 4.8 and 6.2%. The static fields were selected to avoid the transducer and optimized to limit dose to organ at risk limits as shown in the dose volume histograms (DVHs) in Supplementary Fig. 5d. Treatment delivery used standard CBCT-based IGRT followed by delivery of the axial arc. There was no iRAI imaging during VMAT. After the axial arc was treated and the couch rotated 90°, the iRAI was used on the two sagittal static beams as seen in Fig. 5. The two beams were 6-MV X-ray using the flattening filter-free (FFF) mode. The anterior field delivered 141 monitor inferior beam used 187 monitor units at a dose rate of 1,400 monitor units per min.

During the iRAI imaging, the 2D matrix array was held by a homemade mechanical arm, which provided four degrees of freedom. The arm was directly attached to a mobile cart, which carried all the electronic devices, shown in Fig. 5a. To locate the targeted area in the central axis of the field of view, the geometry center of the 2D matrix array was set 4 cm above the isocenter. For acoustic coupling, a water-filled balloon, with its surface applied with ultrasound coupling gel, was directly attached to the surface of the array. The other side of balloon touched the skin of the abdomen with a light pressure. The total distance between the 2D matrix array and the center of target was set to 17 cm.

### Reporting summary

Further information on research design is available in the Nature Portfolio Reporting Summary linked to this article.

## Online content

Any methods, additional references, Nature Portfolio reporting summaries, source data, extended data, supplementary information, acknowledgements, peer review information; details of author contributions and competing interests; and statements of data and code availability are available at 10.1038/s41587-022-01593-8.

## Supplementary information

Supplementary Information Supplementary Figs. 1–5, Discussion and Table 1.

Reporting Summary

Supplementary Video 1 iRAI measured 3D dose distribution of a clinical treatment plan with C-shaped dose distribution.

Supplementary Video 2 iRAI measured temporal dose accumulation of a clinical treatment plan with C-shaped dose distribution.

Supplementary Video 3 iRAI measured temporal dose deposition of a clinical treatment plan with C-shaped dose distribution.

## Data Availability

The authors declare that the data supporting the findings of this study are available within the paper and its supplementary information files. The imaging raw data from the acquisition device are available from University of Michigan Deep Blue Data (10.7302/g05r-5a43).

## Code availability

The codes for data collection and data processing are available from University of Michigan Deep Blue Data (10.7302/g05r-5a43).
