# Supplementary material for: Real-time, volumetric imaging of radiation dose delivery deep into the liver during cancer treatment
Source: Nat Biotechnol. 2023 Jan 2;41(8):1160–7. doi: 10.1038/s41587-022-01593-8 (PMC10314963; doi:10.1038/s41587-022-01593-8)
Supplement: Supplementary file 1 — Supplementary Figs. 1–5, Discussion and Table 1. [file 41587_2022_1593_MOESM1_ESM.pdf]

nature biotechnology

Article

https://doi.org/10.1038/s41587-022-01593-8

Real-time, volumetric imaging of radiation dose delivery deep into the liver during cancer treatment

In the format provided by the authors and unedited

Table of Contents

Supplementary Figures	Fig S1
	Fig S2
	Fig S3
	Fig S4
	Fig S5
Supplementary Table	Table S1
Supplementary Videos	Video S1
	Video S2
	Video S2

Fig S1

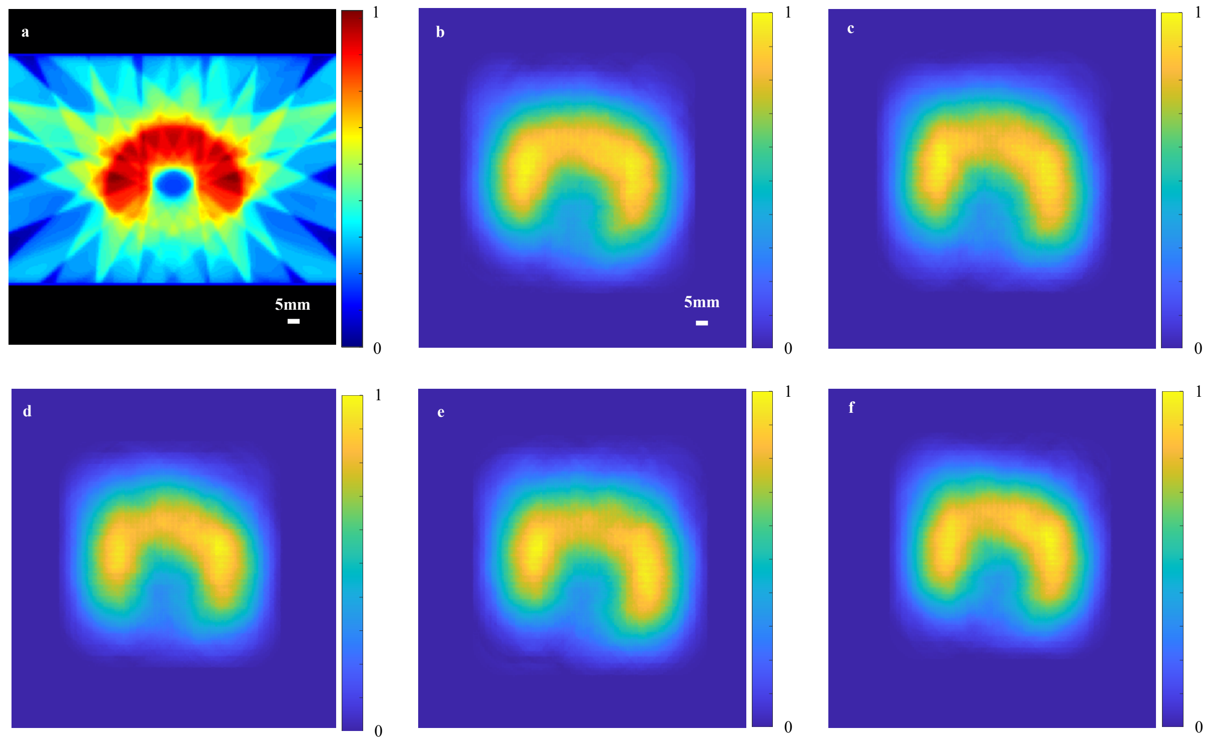

Fig. S1.

Five independent C-shaped relative dose distribution measurements. (A) The planned dose for the C-shaped 3D conformal radiotherapy (CRT) treatment plan. (B-F) iRAI measured results for the C-shaped dose distribution treatment plan.

Fig S2

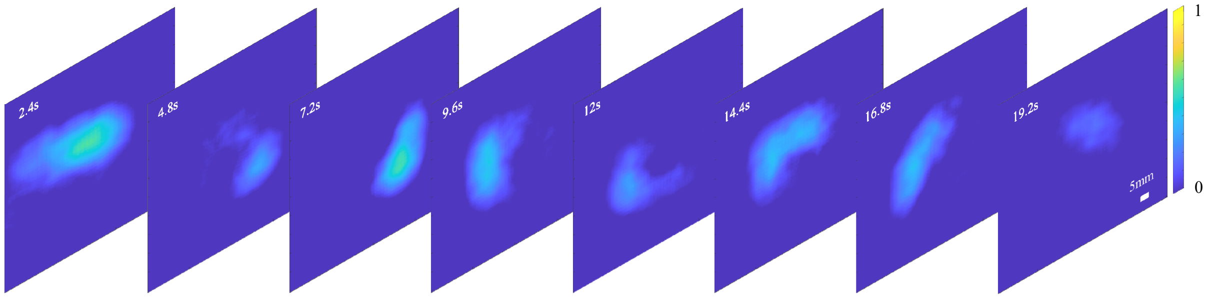

Fig. S2.

The delivered dose of the C-shaped treatment plan between 2 consecutive reconstruction time points measured by iRAI at different time points with 2.4-second interval.

Fig S3

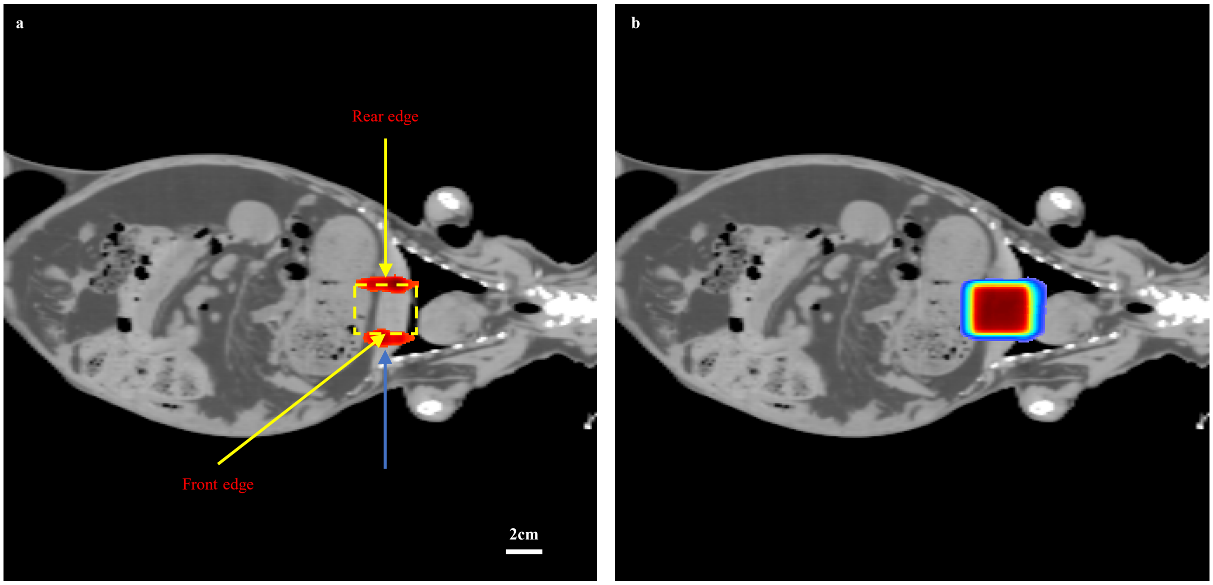

Fig. S3.

In vivo iRAI measurement versus the treatment plan on the coronal plane of a rabbit model with the indication of the front edge and rear edge. (A) The iRAI measurement fused on the CT coronal cross section of the rabbit. (B) The treatment plan fused onto the CT coronal cross section of the rabbit. The yellow dash box indicates the boundary of the treatment plan dose delivery with 5% isodose line. The blue arrow shows the normal direction of the transducer.

Fig S4

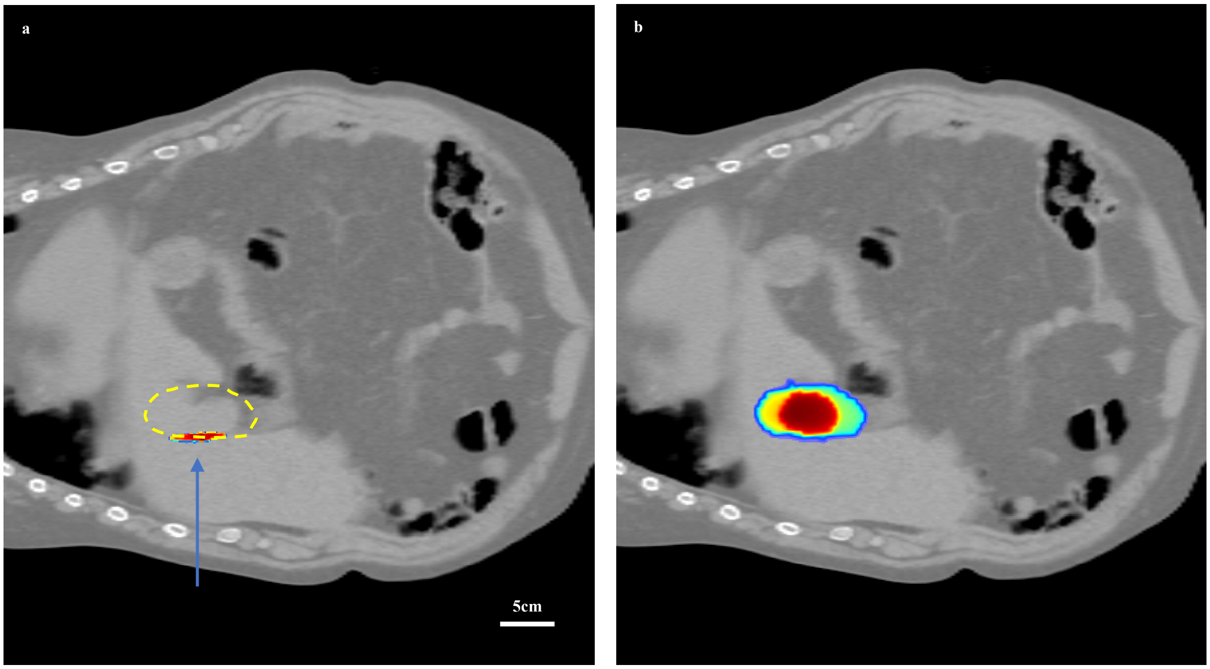

Fig. S4.

In vivo iRAI measurement versus the treatment plan on a coronal plane of a patient. (A) The iRAI measurement fused onto the CT coronal cross section of the patient. (B) The treatment plan fused onto the CT coronal cross section of the patient. The yellow dash area indicates the boundary of the treatment plan dose delivery with 5% isodose line. The blue arrow shows the normal direction of the transducer.

Fig S5

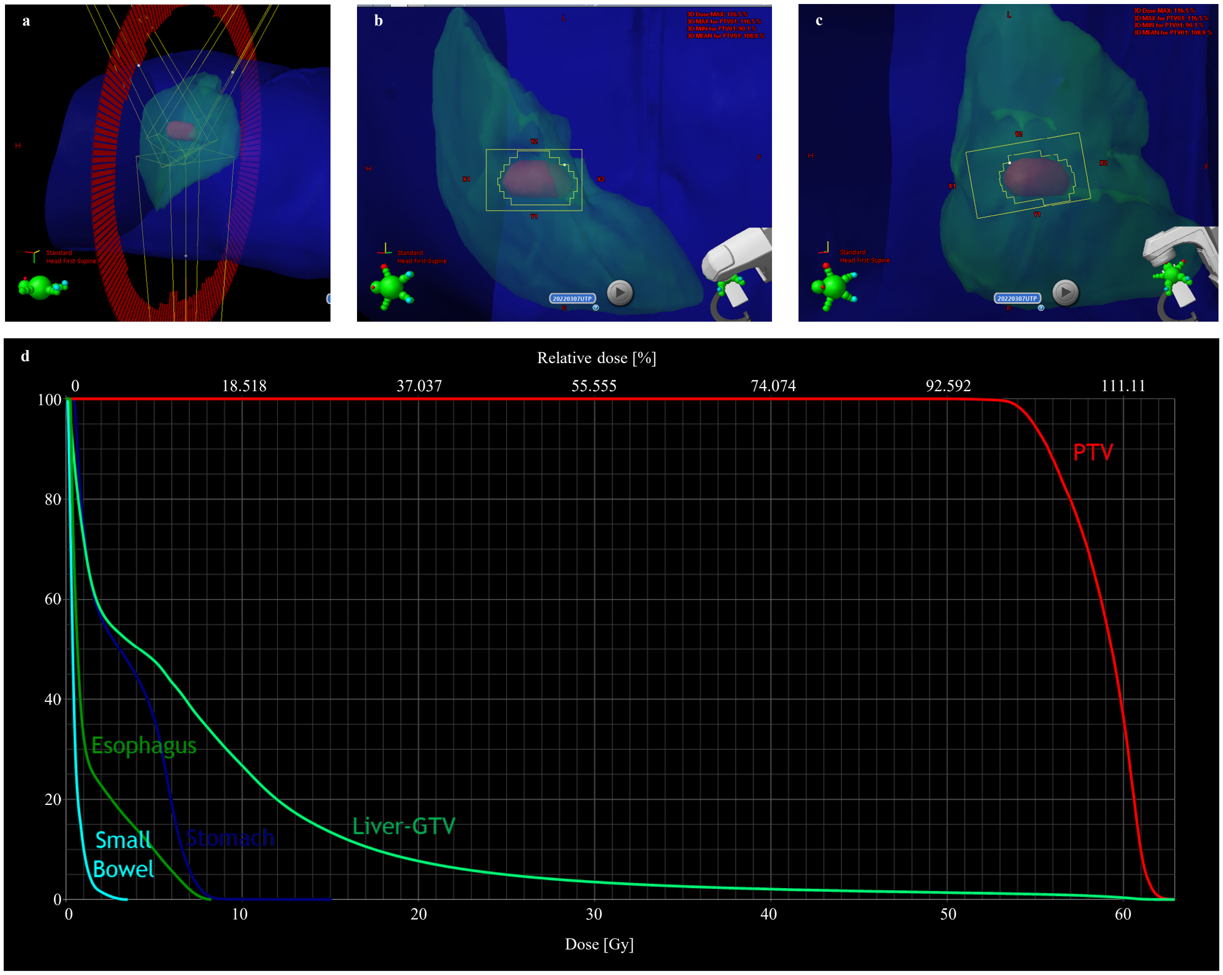

Fig. S5.

The 3D arrangements and the Dose-Volume Histogram (DVH) of the treatment plan for the patient. (A) The whole 3D arrangements of the treatment plan for the patient, including VMAT, superior and inferior anterior beams. (B) The 3D arrangement of the treatment plan for the patient with superior anterior beam. (C) The 3D arrangement of the treatment plan for the patient with inferior anterior beam. (D) The DVH of the treatment plan for the patient meeting clinical constraints.

Table S1.

The RMSE of 3 independent iRAI measurements for the treatment of a rabbit's liver.

RMSE	Measurement 1	Measurement 2	Measurement 3
Front edge	0.0546	0.0581	0.0584
Rear edge	0.0767	0.0701	0.0604

Video S1.
iRAI measured 3D dose distribution of a clinical treatment plan with C-shaped dose distribution.

Video S2.
iRAI measured temporal dose accumulation of a clinical treatment plan with C-shaped dose distribution.

Video S3.
iRAI measured between 2 consecutive reconstruction time points of a clinical treatment plan with C-shaped dose distribution.
