# Supplementary material for: Real-time, volumetric imaging of radiation dose delivery deep into the liver during cancer treatment
Source: Nat Biotechnol. 2023 Jan 2;41(8):1160–7. doi: 10.1038/s41587-022-01593-8 (PMC10314963; doi:10.1038/s41587-022-01593-8)
Supplement: Supplementary file 2 — Reporting Summary [file 41587_2022_1593_MOESM2_ESM.pdf]

nature research

Corresponding author(s): Kyle Cuneo, Xueding Wang, Issam El Naqa

Last updated by author(s): Aug 25, 2022

Reporting Summary

Nature Research wishes to improve the reproducibility of the work that we publish. This form provides structure for consistency and transparency in reporting. For further information on Nature Research policies, see our Editorial Policies and the Editorial Policy Checklist.

Statistics

For all statistical analyses, confirm that the following items are present in the figure legend, table legend, main text, or Methods section.

n/a	Confirmed	
☐	☒	The exact sample size (*n*) for each experimental group/condition, given as a discrete number and unit of measurement
☐	☒	A statement on whether measurements were taken from distinct samples or whether the same sample was measured repeatedly
☒	☐	The statistical test(s) used AND whether they are one- or two-sided<br>*Only common tests should be described solely by name; describe more complex techniques in the Methods section.*
☒	☐	A description of all covariates tested
☒	☐	A description of any assumptions or corrections, such as tests of normality and adjustment for multiple comparisons
☐	☒	A full description of the statistical parameters including central tendency (e.g. means) or other basic estimates (e.g. regression coefficient) AND variation (e.g. standard deviation) or associated estimates of uncertainty (e.g. confidence intervals)
☒	☐	For null hypothesis testing, the test statistic (e.g. *F*, *t*, *r*) with confidence intervals, effect sizes, degrees of freedom and *P* value noted<br>*Give P values as exact values whenever suitable.*
☒	☐	For Bayesian analysis, information on the choice of priors and Markov chain Monte Carlo settings
☒	☐	For hierarchical and complex designs, identification of the appropriate level for tests and full reporting of outcomes
☒	☐	Estimates of effect sizes (e.g. Cohen's *d*, Pearson's *r*), indicating how they were calculated

Our web collection on statistics for biologists contains articles on many of the points above.

Software and code

Policy information about availability of computer code

Data collection	The data was collected by Verasonics Vantage system, which was operated in Matlab 2020a (Mathworks, Inc). The codes of data collection are available from UM Deep Blue Data [https://doi.org/10.7302/g05r-5a43].
Data analysis	The acquired data was processed by Matlab 2020a. All the codes for data processing are available from UM Deep Blue Data [https://doi.org/10.7302/g05r-5a43].

For manuscripts utilizing custom algorithms or software that are central to the research but not yet described in published literature, software must be made available to editors and reviewers. We strongly encourage code deposition in a community repository (e.g. GitHub). See the Nature Research guidelines for submitting code & software for further information.

Data

Policy information about availability of data

All manuscripts must include a data availability statement. This statement should provide the following information, where applicable:

- Accession codes, unique identifiers, or web links for publicly available datasets
- A list of figures that have associated raw data
- A description of any restrictions on data availability

The authors declare that the data supporting the findings of this study are available within the paper and its supplementary information files. The imaging raw data from the acquisition device is available from UM Deep Blue Data [https://doi.org/10.7302/g05r-5a43].

Field-specific reporting

Please select the one below that is the best fit for your research. If you are not sure, read the appropriate sections before making your selection.

☒ Life sciences ☐ Behavioural & social sciences ☐ Ecological, evolutionary & environmental sciences

For a reference copy of the document with all sections, see nature.com/documents/nr-reporting-summary-flat.pdf

Life sciences study design

All studies must disclose on these points even when the disclosure is negative.

Sample size	This study is for in vivo image guidance, which would enable mapping of the delivered radiation without interrupting the current clinical workflow. It is a proof of concept study, which have limited sample size for both animals and patient demonstrating the concept for first time.
Data exclusions	There are few studies used to optimize the imaging system. However, they are not related to the findings of this study.
Replication	At least 3 different measurements were taken for each case to ensure reproducibility. All the attempts at replication were successful.
Randomization	This is proof of concept study for in vivo monitoring of the radiation dose delivery with limited sample size. There is no experiment group or control group. Since we were imaging the radiation delivery to target, the planned doses were used as references.
Blinding	This is proof of concept study for in vivo monitoring the radiation dose delivery. The direct outcome is the imaging results. No control or outcome comparison were included in this study. The only comparison is the imaging results with the treatment plan. At this situation, blinding is not related to this study objectives.

Reporting for specific materials, systems and methods

We require information from authors about some types of materials, experimental systems and methods used in many studies. Here, indicate whether each material, system or method listed is relevant to your study. If you are not sure if a list item applies to your research, read the appropriate section before selecting a response.

Materials & experimental systems

n/a	Involved in the study	
☒	☐	Antibodies
☒	☐	Eukaryotic cell lines
☒	☐	Palaeontology and archaeology
☐	☒	Animals and other organisms
☐	☒	Human research participants
☐	☒	Clinical data
☒	☐	Dual use research of concern

Methods

n/a	Involved in the study	
☒	☐	ChIP-seq
☒	☐	Flow cytometry
☒	☐	MRI-based neuroimaging

Animals and other organisms

Policy information about studies involving animals; ARRIVE guidelines recommended for reporting animal research

Laboratory animals	Two female New Zealand white rabbits (4.5-5kg) with 6 months old, ordered from Charles River, were involved in this study.
Wild animals	The study did not involve wild animals.
Field-collected samples	The study did not involve samples collected from the field.
Ethics oversight	All the animal experiments were approved by University of South Florida Research Integrity and Compliance Institutional Animal Care and Use Committee (IACUC) (Combined radiation acoustics and ultrasound imaging for real-time guidance in radiotherapy, protocol# IS00008026)

Note that full information on the approval of the study protocol must also be provided in the manuscript.

Human research participants

Policy information about studies involving human research participants

Population characteristics	A 60-year-old man diagnosed with liver metastasis treated with radiation therapy was involved in this study.
Recruitment	Physicians specializing in this specific patient care accessed patients during consultation to ensure recruitment strategy is

Recruitment	equitable and represents the population required for the study. Patients will be recruited/offered optional participation in the study at the time of consultation with the physician. Since liver is one of the organs that can be directly affected by respiratory motion and ultrasound imaging compatible, a patient with liver metastasis was recruited in this study. If the organ is not ultrasound imaging compatible, it will be very difficult to detect the radiation acoustic signals.
Ethics oversight	The study was approved by the institutional review board (IRB) of the University of Michigan (UMCC 2017.160 Pilot Study of Combined Radiation Acoustics and Ultrasound Imaging for Guidance in Radiotherapy, HUM00139322). Informed consent was obtained after the nature and possible consequences of the studies were explained. Patient was involved in this study without any participant compensation.

Note that full information on the approval of the study protocol must also be provided in the manuscript.

Clinical data

Policy information about clinical studies

All manuscripts should comply with the ICMJE guidelines for publication of clinical research and a completed CONSORT checklist must be included with all submissions.

Clinical trial registration	No clinical registration involved in the study. This study is for imaging purposes, which was passively detecting the signal without intervention or interruption of the current clinical workflow.
Study protocol	UMCC 2017.160 Pilot Study Of Combined Radiation Acoustics And Ultrasound Imaging For Guidance In Radiotherapy (HUM00139322)
Data collection	The human study data was acquired at the department of radiation oncology, University of Michigan. To minimize the interference for radiation therapy, the treatment plan for each fraction was divided into 2 parts. The first was for for iRAI imaging. The second was a radiotherapy plan to ensure that the total delivered dose met clinical requirements. The patient was setup with the radiotherapy plan first. Then, IRAI device was setup for imaging. The total device setting time and the acquisition time are 5min and 1min, respectively.
Outcomes	The outcome of the measurement was define by SNR of acquired signal from acquisition device. Typically, a SNR higher than 2:1 means the measurement is able to resolve the dose deposition.
